# Therapy Decision Support Based on Recommender System Methods

**DOI:** 10.1155/2017/8659460

**Published:** 2017-03-28

**Authors:** Felix Gräßer, Stefanie Beckert, Denise Küster, Jochen Schmitt, Susanne Abraham, Hagen Malberg, Sebastian Zaunseder

**Affiliations:** ^1^Institut für Biomedizinische Technik, Technische Universität Dresden, Dresden, Germany; ^2^Zentrum für Evidenzbasierte Gesundheitsversorgung, Universitätsklinikum Dresden, Dresden, Germany; ^3^Klinik und Poliklinik für Dermatologie, Universitätsklinikum Dresden, Dresden, Germany

## Abstract

We present a system for data-driven therapy decision support based on techniques from the field of recommender systems. Two methods for therapy recommendation, namely, *Collaborative Recommender* and *Demographic-based Recommender*, are proposed. Both algorithms aim to predict the individual response to different therapy options using diverse patient data and recommend the therapy which is assumed to provide the best outcome for a specific patient and time, that is, consultation. The proposed methods are evaluated using a clinical database incorporating patients suffering from the autoimmune skin disease psoriasis. The *Collaborative Recommender* proves to generate both better outcome predictions and recommendation quality. However, due to sparsity in the data, this approach cannot provide recommendations for the entire database. In contrast, the *Demographic-based Recommender* performs worse on average but covers more consultations. Consequently, both methods profit from a combination into an overall recommender system.

## 1. Introduction

The large volume of daily captured data in healthcare institutions and out-of-hospital settings opens up new perspectives for healthcare. Due to the amount of that data, its high dimensionality and complex interdependencies within the data, an efficient integration of the available information is only possible using technical aids. In this regard, data-driven clinical decision support systems (CDSS) can be expected to take a major role in future healthcare. Generally, CDSS are designated to assist physicians or other health professionals during clinical decision-making. CDSS are demanded to be integrated into the clinical workflow and to provide decision support at time and location of care [1]. Data-driven CDSS, in particular, make use of data-mining and machine-learning techniques to extract and combine relevant information from patient data, in order to provide assistance for diagnosis and treatment decisions or even to be used in clinical quality control based on large-scale data [1].

While most works related to CDSS deal with diagnosis decision support [2, 3], predicting patient condition [4–8], or determining drug interaction [9], data-driven CDSS for therapy decision support are rare to date. This fact can be partially attributed to traditional data-mining and machine-learning techniques, which have limitations in case of missing and inhomogeneous data and often show an undesired black-box behavior.

Within this contribution, we present a system for therapy decision support based on techniques from the field of recommender systems which originates from E-commerce and has developed considerably over the last years. Recommender systems are able to overcome the aforementioned limitations of traditional data-mining and machine-learning techniques, which render suchlike systems an interesting alternative for therapy decision support. In medicine, however, the application of recommender systems is rather limited. In [10], we proposed two methods for therapy recommendation based on recommender systems' techniques, namely, *Collaborative Recommender* and *Demographic-based Recommender.* In this work, we extend our previous work by a comprehensive evaluation of recommenders' performance in terms of accuracy and decision support capability and added a systematic comparison of similarity metrics. Additionally, various aggregation algorithms are compared differing in the way how similarity between consultations, that is, patients and their overall therapy response impacts the therapy recommendations. Finally, in extension to [10], a possible fusion approach is introduced for combining both individual recommendation engines. The proposed methods are evaluated using a clinical database incorporating patients suffering from the autoimmune skin disease psoriasis.

## 2. Background and Basic Taxonomies

### 2.1. Clinical Decision Support Systems

In general, CDSS can be classified into knowledge-based and data-driven approaches having both advantages and suffering from disadvantages. Knowledge-based systems on the one hand usually rely on manually encoded rule-based expert knowledge (if-then rules) to infer decision support. Applied rules typically represent clinical guidelines and best practice rules providing a reliable decision basis [11–13]. Disadvantage of such approaches is the bottleneck during development and updating on the basis of population-based studies and limited personalization.

Data-driven approaches on the other hand apply methods from data-mining and machine-learning to automatically extract knowledge from clinical data, facilitating more individual recommendations, learning from past experience, and revealing unknown patterns in the available data [14]. Clinical data, however, is characterized by uncertainties as well as heterogeneity (various data types), high dimensionality, and incompleteness (sparsity) [14, 15]. Such structure and characteristics put challenges on conventional machine-learning methods such as support vector machines, artificial neural networks, and decision trees. Though some conventional machine-learning techniques can cope with suchlike data properties, they require application of problem-specific a priori knowledge and highly complex models. Additionally, a crucial disadvantage of such methods in the context of CDSS is the limited interpretability (black-box behavior) of the produced results, which leads to lacking acceptance amongst health professionals. Recommender systems can overcome the limitations of traditional data-mining or machine-learning techniques, which makes them a highly interesting choice even for medical applications.

### 2.2. Recommender Systems and Intended Use

Recommender system technologies date back to the nineties [16–18] and are primarily intended to make personalized product suggestions based on previously recorded data on users' preferences [19]. Nowadays, recommender systems are an accepted and widespread technology used by many market leaders of various industries (e.g., Amazon (https://www.amazon.com), Netflix (https://www.netflix.com), and Spotify (https://www.spotify.com)).

Over the years, the field of recommender systems has evolved considerably yielding extremely sophisticated and specialized methods depending on domain, purpose, and personalization level [20]. Unlike conventional machine-learning methodologies, recommender systems can be capable of coping with the stated challenges associated with the data to be processed and are additionally able to permit insight into the decision-making process making results interpretable. Surprisingly, recommender systems have not found wide application in medicine so far.

A basic taxonomy of recommendation algorithms differentiates between content-based [21], collaborative filtering [19], and hybrid approaches [22]. All approaches have in common to convert estimations of a user's preference for items into recommendations using explicit or implicit previous ratings as expressions of preference. While the content-based approach links preference to item attributes, the collaborative filtering method considers the ratings of other users in the system to make personalized predictions on an active user's preference. The underlying algorithms for rating prediction and recommendation computation are based on similarity metrics which are capable of processing sparse and inhomogeneous data vectors. Furthermore, by presenting the respected data subset and impact factors, interpretation and explanation of the recommendation can be provided. Finally, the underlying databases of such systems can easily be adapted and extended which correspond to continuous adaption to new impact factors and environment in which the system is applied [20, 23].

### 2.3. Approaches for Therapy Decision Support

Concerning data-driven therapy or treatment recommendation in general, some scientific works were proposed, ranging from approaches based on majority voting [24], systems based on association rules [25], or applying case-based reasoning [26]. In spite of the named benefits, as stated beforehand, the application of recommender system methods in the medical context is generally limited, and there is mainly work loosely related to the idea of using suchlike methods for therapy decision support. Proposed medical applications of recommender systems are presentation of personalized health record related information for both patient and medical practitioner [27], optimized literature search for radiologists, and disease risk or mortality prediction [6, 28, 29]. Using typical recommender system methodologies for treatment recommendation, two related works are a nursing care plan [30] and an approach recommending wellness treatment [26], both applying collaborative filtering techniques.

In this work, we transfer the idea of collaborative filtering to the domain of CDSS. We present a recommender system which aims at predicting the adequacy of different therapy options for a given patient at a given time. To that end, two methodologies for therapy adequacy estimation, a *Collaborative Recommender* and a hybrid *Demographic-based Recommender*, are compared and finally combined to an ensemble of recommenders aiming at compensating for the individual algorithms' drawbacks. The exemplary recommender system is developed targeting therapy recommendations for patients suffering from the skin disease psoriasis.

## 3. Materials and Methods

### 3.1. Data

In this work, different recommender system approaches are developed and evaluated based on excerpts from health records provided by the Clinic and Polyclinic for Dermatology, University Hospital Dresden. The data consists of *V* = 1111 consultations from 213 patients suffering from various types of psoriasis. For each of the consultations, patient and therapy describing attributes are at hand, containing demographic data, information on comorbidities, and state of health as well as previous and current local and systemic treatment. The data was manually extracted from health records and stored in a MySQL database. Within a careful revision process, corrupted and invalid data was corrected or eliminated. Overall, the data comprises *A*_0_ = 20 patient describing attributes for each consultation and *T* = 3 therapy describing attributes for each of the therapies applied. However, some patient describing attributes were converted into binary features for further processing which extends the patients describing feature space to *A* = 123 features.

The different attributes making up the data are of various levels of measurement ranging from dichotomous to ratio-scaled attributes. Moreover, in spite of data padding in cases where information was missing but could be assumed to be constant over consultations, availability of certain attributes is very limited. As a consequence, the resulting data matrices are characterized by inhomogeneity and sparsity. Patient attributes and therapy information are summarized in Tables 1 and 2 along with scale of measurement, range of values, and relative availability, respectively. Attributes that were present in less than five percent of the available data were neglected.

Previous treatment is the collection of all relevant therapies applied to a patient up to the consultation under consideration, whereas in the current treatment database, all therapies are collected which were applied within the last two weeks preceding the respective consultation. Even though there is information on both local and systemic therapies available, this study focuses on recommending the most effective systemic therapy out of *M* = 15 available therapies of this type for a given consultation. For both previous and applied therapies, up to three outcome indicators are intermittently given: (1) a therapy effectiveness indicator (bad, medium, and good), representing the subjective assessment, (2) an objective health state improvement indicator, and (3) adverse effects (yes, no). The health state improvement indicator relates to the severity of psoriasis quantified by the Psoriasis Area and Severity Index (PASI). PASI ranges from 0 (no disease) to 72 (maximal disease) combining both the skin area affected and the severity of lesions in a single score [31]. Change of PASI between two consecutive consultations (ΔPASI) is assigned as objective health state improvement indicator attribute to all therapies applied between those consultations where the change occurred.

### 3.2. Methodology

#### 3.2.1. System Overview

The algorithms described in the following aim at recommending the potentially most effective systemic therapy for a given patient and consultation. The collaborative filtering idea is transferred to the therapy recommendation domain, considering therapies as items and therapy response as a user's preference. For representing therapy response, effectiveness, ΔPASI, and absence of adverse effects are incorporated.

In a preceding prediction step, individual therapy outcome is estimated for all available therapies that have not yet been applied to the patient. The outcome estimate is computed based on the therapy response of the nearest neighbors to the consultation under consideration. At this stage, similarity computation between consultation representations plays an essential role. The two recommender approaches proposed in this work differ in the information used to represent consultations. The applied *Collaborative Recommender* algorithm uses solely outcome from all preceding consultations to represent a consultation. The hybrid *Demographic-based Recommender* approach on the other hand is taking additionally all available patient describing data into account. In the subsequent recommendation step, the therapies are ranked according to their response estimates and the top *N*-ranked entry or entries are recommended. Both recommender engines suffer from drawbacks depending on the data properties which the other approach is capable of compensating for. Therefore, an ensemble of recommenders is introduced combining both recommender engines. In the following sections, computation of therapy response estimates is detailed, both recommender approaches along with the applied similarity metrics are described and the actual outcome prediction algorithm is presented. Finally, the ensemble model is described and the applied evaluation metrics are introduced.

#### 3.2.2. Affinity Model

**Figure 1:**
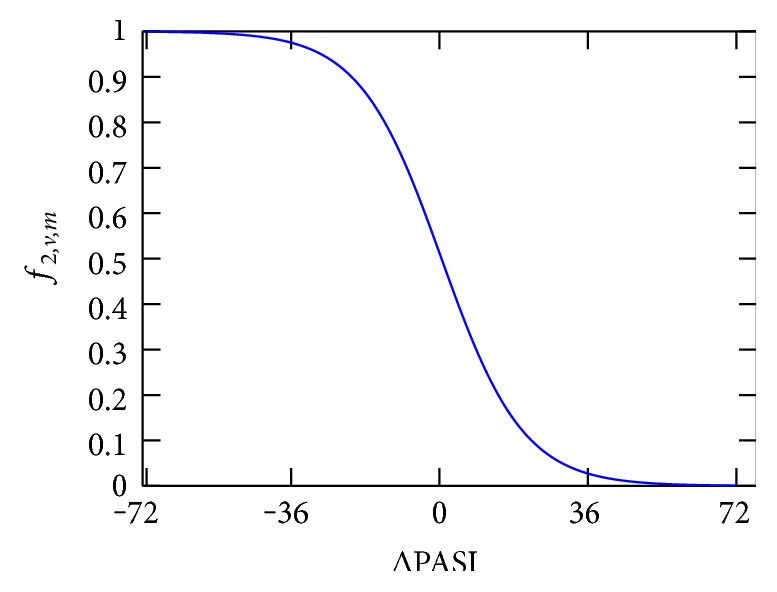
Sigmoid function mapping the ΔPASI score to the domain  0 ≤ *f*_*t*,*v*,*m*_ ≤ 1.

In typical recommender system applications, data reflecting a user's preferences is collected from both explicit and implicit input. Where explicit expression of preference usually is provided as item ratings or votes on items, implicit information can be derived from clicked items, items being part of the shopping basket, or visited pages, respectively. Here, the preference to a therapy is derived from the therapy response. The mathematical quantification of therapy response, in the following denoted as *affinity*, is modeled using a weighted sum of three parameters attained from subjective effectiveness (*f*_1,*v*,*m*_), change of the PASI score (ΔPASI), that is, current PASI compared to the PASI of the previous consultation (*f*_2,*v*,*m*_), and adverse effects (*f*_3,*v*,*m*_). The individual components impact on the overall affinity measure can be varied by adjusting weight *w*_*t*_. As was figured out in own previous analysis, particularly the *Collaborative Recommender* approach benefits from decreasing influence of adverse effects. Consequently, the adverse effect weight was set to *w*_3_ = 0.25, whereas both effectiveness indicators have equal weight *w*_1/2_ = 1. As can be seen in Table 2, the three attributes are just intermittently available, meaning that not all applied therapies are provided with all three components. Missing data is respected by normalization with the sum of weights *w*_*t*_, where *δ*_*t*,*v*,*m*_ is set to 0 in case of missing values and set to 1 otherwise. Thus, the affinity of a given patient and consultation *v*  ∈  *V* to a therapy *m*  ∈  *M* is modeled as

(1) $$\begin{array}{c} r_{v,m}=\frac{\sum_{t=1}^{T}\delta_{t,v,m}\cdot w_{t}\cdot f_{t,v,m}}{\sum_{t=1}^{T}\delta_{t,v,m}\cdot w_{t}}, \end{array}$$

where the two effectiveness related affinity components (subjective effectiveness and ΔPASI score) are mapped to the domain 0 ≤ *f*_*t*,*v*,*m*_ ≤ 1 according to the following specifications. Effectiveness is factorized with a constant value resulting in the nominal values 0.25 (poor), 0.5 (moderate), and 0.75 (good). The ΔPASI score is mapped by a negative sigmoid function adjusted to the domain 0.1 as shown in Figure 1 facilitating large impact on small PASI score variations declining with increasing absolute value. Finally, the binary adverse effect indicator is mapped to −0.25 if any adverse effect is present and 0 otherwise to penalize the overall affinity measure if adverse effects have occurred. The mapping rules can be summarized as

(2) $$\begin{array}{c} f_{1,v,m}=0.25\cdot effectiveness, \end{array}$$

(3) $$\begin{array}{c} f_{2,v,m}=1-\frac{1}{1+e^{-0.1\cdot \Delta PASI}}, \end{array}$$

(4) $$\begin{array}{c} f_{3,v,m}=\left\{\begin{array}{ll} -0.25 & if\;adverse\;effect\\ 0 & otherwise \end{array}\right.. \end{array}$$

#### 3.2.3. Similarity Metrics

Both proposed methods, *Collaborative Recommender* and *Demographic-based Recommender*, are related to user-user collaborative filtering [20] which correlate between consultations as users to recommend therapies as items. The basic idea is to make affinity estimations for (a subset of) therapies which were not yet applied to a patient. For affinity estimation, a neighborhood-based algorithm is used [32]. Therefore, similarity computation between consultations has critical impact on the recommender engine's output and is highly dependent on the consultation representation. Similarity *w*_*v*,*k*_ between two consultations *v*  ∈  *V* and *k*  ∈  *V* is calculated for all consultation pairs considering only therapies *m*  ∈  *M* which are corated in both consultations. Depending on the information which is used for consultation representation, a distinction between the two aforementioned recommendation techniques and consequently the similarity metrics applied can be made. In this section, various similarity metrics are detailed which were studied in this work for both, the *Collaborative Recommender* approach and *Demographic-based Recommender* approach.

*(1) Collaborative Recommender.* In this approach, the consultation under consideration is only represented by the affinity values related to therapies applied up to this consultation. The underlying assumption is that the therapy applied to a given patient within the therapy history and the associated outcome reincorporates information about that respective patient and consultation which can then be transferred to patients with similar therapy history. Here, all attributes respected for similarity computation, that is, affinity entries for previously applied therapies, are of ratio scale and within the same range of values. However, the affinity matrix is characterized by only intermittently available entries, that is, resulting in sparse data vectors to be compared. Three similarity metrics proposed in the recommender system literature [32] are investigated and compared in this work. *Vector Similarity*,

(5) $$\begin{array}{c} w_{v,k}^{1}=\frac{\sum_{m\in M}r_{v,m}\cdot r_{k,m}}{\sqrt{\sum_{m\in M}r_{v,m}^{2}}\cdot \sqrt{\sum_{m\in M}r_{k,m}^{2}}}, \end{array}$$

which originates from information filtering using vector space models, is widely used in collaborative filtering algorithms. Vector Similarity simply computes the cosine of the angle between two vectors *r*_*v*,*m*_ and *r*_*k*,*m*_ representing two consultations *v*  ∈  *V* and *k*  ∈  *V*, respectively. Furthermore, the degree of linear relationship between two vectors can be quantified using the *Pearson Correlation*,

(6) $$\begin{array}{c} w_{v,k}^{2}=\frac{\sum_{m\in M}\left(r_{v,m}-\bar{r_{v}}\right)\cdot \left(r_{k,m}-\bar{r_{k}}\right)}{\sigma_{v}\cdot \sigma_{k}}, \end{array}$$

derived from a linear regression model. This metric relies on the assumption that a linear relationship must exist and the errors are independent and have a probability distribution with zero mean and constant variance. However, these assumptions are often violated in the context of collaborative filtering data which can deteriorate the outcome accuracy. To overcome these stated model assumptions, the *Spearman Rank Correlation*,

(7) $$\begin{array}{c} w_{v,k}^{3}=\frac{\sum_{m\in M}\left({rank}_{v,m}-\bar{{rank}_{v}}\right)\cdot \left({rank}_{k,m}-\bar{{rank}_{k}}\right)}{\sigma_{v}\cdot \sigma_{k}}, \end{array}$$

computes a measure of correlation between ranks of the individual therapies' affinity values instead of the affinity values directly.

**Figure 2:**
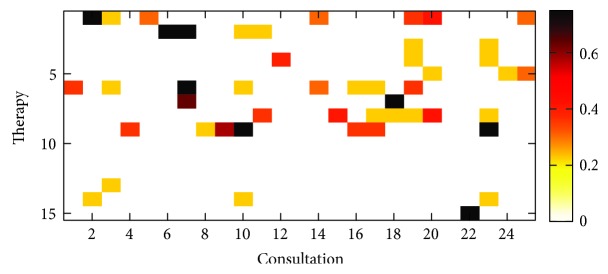
Affinity matrix for 25 randomly selected consultations.

Figure 2 depicts an exemplary affinity matrix excerpt. Here, the affinity measure derived from the available information on therapy outcome from 25 randomly selected consultations is shown. As can be seen, only a small fraction of the 15 available therapies were applied per patient and consultation. Additionally, only a reduced number of therapy preference parameters are available for those applied therapies. As stated beforehand, this results in an extremely sparse affinity matrix for consultation representation which is relied upon when computing similarity. Additionally, this approach suffers from the so-called *cold start* problem occurring when a new patient is included into the system providing no therapy history at all. However, lacking information makes it difficult or even impossible to find appropriate similar consultations [20].

One approach to address the trust that can be placed in the similarity to a neighboring consultation, depending on the available information, is significance weighting. In case of the *Collaborative Recommender* approach, a significance weight depending on the number of co-rated therapies *n* (out of *N*) included in the computation between consultations is applied. Thus, if two consultations to be compared have fewer than *b* therapies (affinity entries) in common, the similarity weight is decreased, respectively. However, results showed that the *Collaborative Recommender *is not benefiting from significance weighting in this application.

(8) $$\begin{array}{c} w_{v,k}=\left\{\begin{array}{ll} \frac{n}{N}\cdot w_{v,k} & n\leq b\\ w_{v,k} & otherwise. \end{array}\right.. \end{array}$$

*(2) Demographic-Based Recommender.* To overcome the limitations related to the above described collaborative filtering approach concerning lacking information and cold start, the *Collaborative Recommender* is extended to utilizing all patient describing information summarized in Table 1 to represent a consultation. The straightforward underlying assumption here is that the available patient describing data carries sufficient information for facilitating meaningful comparisons between consultations. However, as already stated in Section 3.1, the patient describing data employed for consultation comparison is not only sparse but the attributes involved into the similarity calculation are characterized by inhomogeneity, that is, are of various level of measurement (dichotomous, nominal, ordinal, interval, and ratio scaled). The similarity measure utilized in this work facilitating both, handling missing values and varying levels of measurement, is the *Gower Similarity Coefficient* [33]. Here, the level of measurement of the individual attributes is respected for each attribute comparison. Furthermore, the Gower coefficient offers the opportunity to control the individual attribute's impact on overall similarity by assigning specific weights to attributes. Thus, the overall similarity *w*_*v*,*k*_ between two consultations is computed including the individual attribute similarities *ρ*_*a*,*v*,*k*_ depending on their presence *δ*_*a*,*v*,*k*_ and assigned weights *w*_*a*_ as

(9) $$\begin{array}{c} w_{v,k}^{4}=\frac{\sum_{a\in A}\delta_{a,v,k}\cdot w_{a}\cdot \rho_{a,v,k}}{\sum_{a\in A}\delta_{a,v,k}\cdot w_{a}}. \end{array}$$

Finally, the computed overall similarity is normalized with the sum of weights *w*_*a*_ of present values by setting the *δ*_*a*,*v*,*k*_ to 0 in case of missing values and 1 otherwise, respectively. The data type-specific similarity coefficients *ρ*_*a*,*v*,*k*_ are defined as follows: For similarity computation between ordinal, interval, and ratio-scaled values, the Manhattan distance normalized to the attribute range is utilized, whereas for nominal or dichotomous attributes, simple matching (*M*-coefficient) or the Jaccard similarity (*S*-coefficient) coefficients are applied, respectively.

Significance weighting in case of the *Demographic-based Recommender* showed slight improvement over the unweighted similarity between consultations. Here, similarity is penalized in case of fewer than *b* = 30 common attributes from the extended feature space *A*.

#### 3.2.4. Affinity Estimation

To generate an affinity estimate on appropriate therapies for a consultation under consideration, various methods for computing aggregates of neighboring consultations' therapy response, that is, affinity, are compared. Here, an affinity estimate *r*_*v*,*m*_ for a consultation under consideration *v*  ∈  *V* and therapy *m*  ∈  *M* is calculated according to three different calculation rules, differing in the way the affinity distribution is accounted for. When computing the affinity prediction, previous consultations of the same patient need to be discarded. The basic way for affinity estimation is computing a simple average over all *K* most similar consultations *k*  ∈  *K*

(10) $$\begin{array}{c} p_{v,m}^{1}=\frac{\sum_{k\in K}r_{k,m}}{\left|K\right|} \end{array}$$

which serves as baseline estimate. To account for the similarity between consultations and to control influence on the outcome, the above formula is extended estimating affinity as weighted average

(11) $$\begin{array}{c} p_{v,m}^{2}=\frac{\sum_{k\in K}r_{k,m}\cdot w_{v,k}}{\sum_{k\in K}\left|w_{v,k}\right|} \end{array}$$

assuming that all consultation affinity entities have the same distribution. Under the assumption, that therapy outcome for different consultations, that is, patients, is centered around different means, the weighted average of deviations from the neighboring consultations' means is computed. Thus, the affinity for a consultation under consideration and therapy is determined by adding this average deviation across all respected neighboring consultations to the consultation under consideration's mean affinity

(12) $$\begin{array}{c} p_{v,m}^{3}=\bar{r_{v}}+\frac{\sum_{k\in K}\left(r_{k,m}-\bar{r_{k}}\right)\cdot w_{v,k}}{\sum_{k\in K}\left|w_{v,k}\right|}. \end{array}$$

For all introduced approaches, the summations are performed over the *K* most similar consultations *k*  ∈  *K* as predictors for therapy *m* with *w*_*v*,*k*_ being the weight between consultations *v* and *k*, representing similarity, and *r*_*k*,*m*_ being the affinity during consultation *k* on therapy *m*. The size of an adequate subset of consultations, that is, the number of nearest neighbors *k* included in the computation, is crucial and needs to be chosen cautiously. Two approaches are possible for the application at hand. Correlation thresholding on the one hand determines the respected neighborhood size according to a predefined threshold assuming that highly correlating consultation are more valuable predictors. However, in this work, the best *K* neighbors approach is applied which considers a predefined number *K* of predictor consultations as consultation subset size. Both approaches suffer from reduction in performance and prediction quality due to noise when too many consultations are included into the estimate computation. In contrast, having too few neighbors for affinity estimation, the coverage of available therapies can be very low.

#### 3.2.5. Recommender Ensemble

As stated beforehand, both proposed recommender approaches have their strengths and weaknesses. The idea of building a recommender ensemble is to generate an overall recommendation which combines both approaches while compensating for the individual recommender engines' drawbacks. Fusing decisions in machine-learning applications, denoted as ensemble learning, have shown to be capable of outperforming basic algorithms [34]. The extensive benefit of decision fusion has also been proven in the Netflix Grand Prize in 2009 where a combination of algorithms finally achieved the largest accuracy improvement (http://www.netflixprize.com). In the contrast to fusion of two competitive recommender engines as proposed in [35] to benefit from as much information as possible, selecting one recommender assumed to be expert under certain constraints is applied here to overcome the stated cold start problem.

### 3.3. Evaluation

In this work, two different evaluation metrics are considered. On the one hand, the individual recommender engine or system yields to predict the response to specific therapies. If the prediction meets the real therapy response, the system can provide the medical practitioner with a reliable support for his decision-making based on the estimation. To quantify the difference between estimated response and real response, the root mean squared error (RMSE) for a specific consultation is computed between provided affinity entries and predictions. RMSE reflects the rating error in the same value domain as the actual affinity measure with large errors having more impact [32].

(13) $$\begin{array}{c} {RMSE}_{v}=\frac{1}{M_{v}}\sqrt{\sum_{m=1}^{M_{v}}{\left(p_{v,m}-r_{v,m}\right)}^{2}}. \end{array}$$

On the other hand, *N* top-ranked therapies are usually selected from the affinity predictions for a consultation under consideration and presented to the user. For evaluating recommendation quality, decision support accuracy metrics are commonly utilized as described in [36]. In our application, the objective is to evaluate how effective the recommender engine or system is at helping a medical practitioner to select an appropriate therapy from the set of all therapies which is essential for boosting acceptance of suchlike systems. The challenge in the context of therapy recommendation is the only partially observed ground truth as described in [37]. Thus, an outcome-driven precision metric as additional decision support metric is defined as follows. Initially, all consultations are divided into those cases where at least one of the top *N* therapies recommended were actually applied in the respective consultation and those where the therapies were not compliant. Furthermore, therapies were considered having good outcome if the affinity assigned to that therapy complies with *r*_*v*,*m*_ ≥ 0.5 and *r*_*v*,*m*_ < 0.5 otherwise which leads to the definitions summarized in Table 3.

The outcome-driven precision describes the ratio of all therapies recommended by the system for a consultation *v*  ∈  *V*, that is, top *N* therapies, which were applied and show good response, that is, are considered successful, and is defined as

(14) $$\begin{array}{c} Precision@N_{v}=\frac{{TP}_{v}}{{TP}_{v}+{FP}_{v}}. \end{array}$$

## 4. Results and Discussion

**Figure 3:**
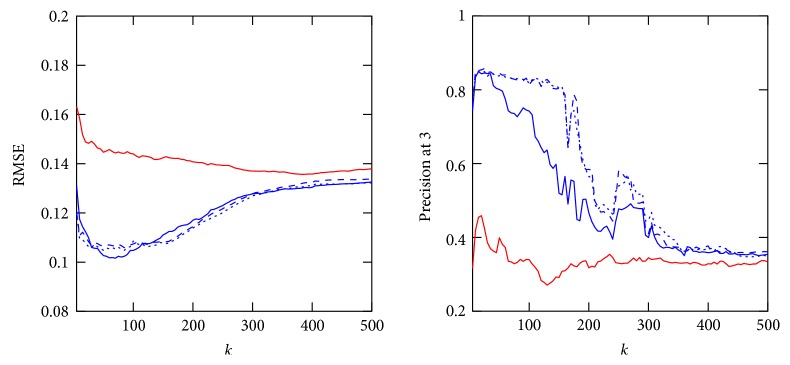
RMSE and precision at 3 over 5 ≤ *k* ≤ 500 of *Demographic-based Recommender* and *Collaborative Recommender* employing Gower (red line), cosine (blue dash line), Pearson correlation (blue dotted line), and Spearman correlation (blue line) for similarity computation and averaging for affinity estimation.

**Figure 4:**
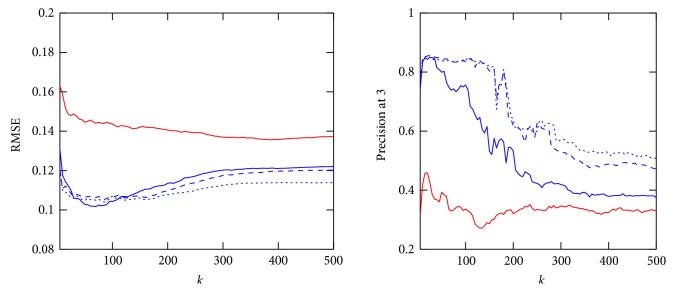
RMSE and precision at 3 over 5 ≤ *k* ≤ 500 of *Demographic-based Recommender* and *Collaborative Recommender* employing Gower (red line), cosine (blue dash line), Pearson correlation (blue dotted line), and Spearman correlation (blue line) for similarity computation and weighted averaging for affinity estimation.

**Figure 5:**
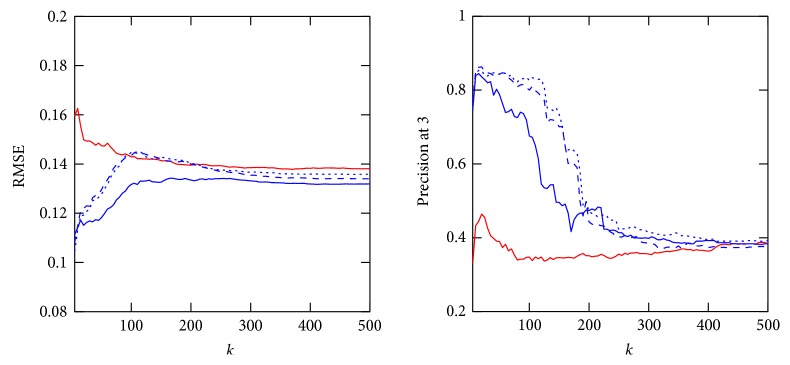
RMSE and precision at 3 over 5 ≤ *k* ≤ 500 of *Demographic-based Recommender* and *Collaborative Recommender* employing Gower (red line), cosine (blue dash line), Pearson correlation (blue dotted line), and Spearman correlation (blue line) for similarity computation and normalized weighted averaging for affinity estimation.

**Figure 6:**
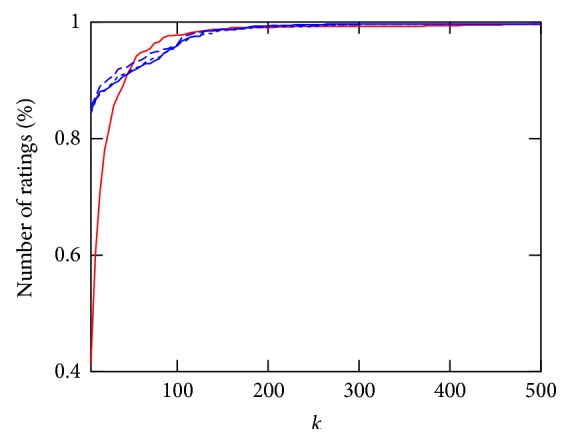
Relative number of consultations for which affinity estimation RMSE can be computed depending on 5 ≤ *k* ≤ 500 for *Demographic-based Recommender* and *Collaborative Recommender* employing Gower (red line), cosine (blue dash line), Pearson correlation (blue dotted line), and Spearman correlation (blue line) for similarity computation.

In the following, the three affinity estimation approaches introduced in Section 3.2.4 are compared for both the *Collaborative Recommender*, employing all three proposed similarity metrics, and the *Demographic-based Recommender.* Figures 3, 4, and 5 demonstrate affinity estimation error and recommendation precision for all proposed affinity prediction algorithms. Both evaluation metrics are averaged over all consultations for each distinct number of the nearest neighbors *k* being evaluated. In case of the *Collaborative Recommender* approach, the estimated affinity can only be compared, that is, an estimation error computed, if (1) therapy describing data from previously applied therapies is available. Without such information no similarity and consequently no affinity estimate and recommendation can be computed. (2) Therapy describing data for therapies applied in the consultation under consideration are available. The ratio of consultation for which both requirements are met amounts to 76.87% of all consultations in the database. Furthermore, prerequisite for affinity estimate evaluation, that is, RMSE computation, are common therapies for which affinity entries are available in both estimation and applied therapy vector. The relative number of consultations for which this requirement is met and the RMSE is computed from increases with the size of the neighborhood *k* as demonstrated in Figure 6 for both recommender approaches.

Regarding recommendation precision, the ground truth is obtained from all consultations having one or more therapies which showed good response according to the definition described in Section 3.3. As a result, precision can only be computed for 67.24% of all consultations in the database for the *Demographic-based Recommender* and 61.48% for the *Collaborative Recommender* approach.

In both nonnormalized *Collaborative Recommender* approaches, the affinity estimation error quickly declines for all studied similarity metrics with increasing *k*. Both approaches have a minimum at around *k* = 75 considered nearest neighbors. The *Demographic-based Recommender* affinity prediction error is significantly higher. However, the *Demographic-based Recommender's* performance improves asymptotically with rising *k*. Concerning recommendation precision, both nonnormalized *Collaborative Recommender* approaches perform very robust on up to around 150 nearest neighbors for cosine similarity and the Pearson correlation. The *Demographic-based Recommender* again provides substantially lower precision with a maximum at a very small neighborhood of around *k* = 20. In case of the *Collaborative Recommender*, the Spearman correlation for computing similarity between consultations is able of having a minor advantage when estimating affinity, but the recommendation precision is only for a very small *k* as good as the other similarity metrics. Particularly Pearson correlation clearly shows the advantage of weighting the impact of neighbors according to their similarity compared to the nonweighted averaging approach. By considering similarity as weight, the influence of noise introduced by more distant neighbors is reduced resulting in smaller prediction errors and better recommendation precision for large neighborhoods. However, the nonweighted averaging approach approximates asymptotically the mean affinity for each of the therapies as affinity estimate for large *k* and yields only low precision. Normalization by adding deviations from average affinity for each respected neighbor to the mean affinity for each consultation under consideration is not improving the computed estimate. This is mainly due to the fact that average is computed here for only a very small number of applied therapies having no representative meaning on overall outcome tendency for a consultation under consideration.

As stated beforehand, for the *Collaborative Recommender* affinity estimation and consequently therapy recommendation can only be generated if therapy describing data from previous therapies is available for neighborhood selection. In the provided data, only 85.33% of all consultations are applied with such data from previous therapies. To overcome this cold start limitation, the *Demographic-based Recommender* can adopt the recommendation task for the remaining 14.67% consultations being not dependent on historical therapies and outcome information. In the following, for all consultations providing previous therapy information, recommendations are computed using the weighted averaging *Collaborative Recommender* approach. For similarity computation, Pearson correlation is employed yielding the best precision performance in the neighborhood of *k* = 100 neighbors. All recommendations which could not be provided by the *Collaborative Recommender* due to missing information were imputed by a weighted averaging *Demographic-based Recommender* showing the best performance with *k* = 20. This approach is capable of compensating the deficiency in recommendations due to lacking information resulting in recommendations for all consultations in the available data. Using this ensemble approach, a mean precision over all consultations of 79.78% could be obtained. In comparison, utilizing average affinity for each therapy in the entire database to determine recommendations to compensate the *Collaborative Recommender* gaps, that is, recommending the therapies ranked by their mean response, overall precision of 78.51% is yielded.

## 5. Conclusion

In this work, the application of recommender system algorithms in the context of therapy decision support was studied. Even though there is an extensive impact of recommender systems in other domains, application of suchlike approaches in healthcare are—to the best of our knowledge—still rare to date. Dependent on the data employed for determining similarity between consultations and therapy outcome estimation, two approaches were compared. For both algorithms, a *Collaborative Recommender* approach and *Demographic-based Recommender*, various variations were studied concerning similarity metric, considering credibility of the computed similarity and aggregation of the respected information for estimating potential therapy response. All algorithms were evaluated using the accuracy of predicting the outcome and according to the precision with which the top 3 recommended therapies meet the ground truth. However, as ground truth only therapies were accepted which have shown good response, therapies with bad outcome were neglected. Therefore, evaluation could only be done on a fraction of the already rather limited database. However, it is assumed that the performance of the approaches studied in this work depend substantially on the amount of available data and will improve considerably if scaled to larger datasets.

The *Collaborative Recommender* utilizing basic collaborative filtering algorithms, considering only therapy outcome from previously applied therapies for consultation representation, outperforms the *Demographic-based Recommender* approach. The weighted averaging *Collaborative Recommender* method taking the similarity weight into account demonstrates better performance than simple averaging over all neighborhood sizes studied in this work. Normalization with respect to deviations from average response for individual consultations performs significantly worse. Concerning similarity metrics, the Pearson correlation shows the best results by exceeding both the cosine similarity and the Spearman rank correlation especially with increasing size of the respected neighborhood.

Applying patient describing data for similarity computation, that is, the *Demographic-based Recommender* approach, does not show a comparable performance to the *Collaborative Recommender*. The similarity computation underlying the *Demographic-based Recommender* is affected unfavorably by less relevant information included into the calculation whereas more important factors have too little effect. Therefore, future work will concentrate on improving the *Demographic-based* method using features selection and weighting methods and learning optimized similarity metrics. Including more information into the recommendation is essential to overcome the limitations in cases where no data on therapy history or just little information is available for a specific patient under consideration as was demonstrated. A simple combination of both recommender approaches was generated which replace each other depending on the available information. Therewith, the cold start problem could be overcome and recommendations provided for consultations having no information on therapy history. In future work, more sophisticated hybrid [22], time-aware approaches considering feature and preference evolution [38], and recommender ensembles will be studied incorporating information from both approaches into the entire recommendation process.

Further on, beyond the presented comparison of different recommender-based approaches, a comparison of the proposed methods to alternative machine-learning algorithms for generating therapy recommendations, particularly model-based approaches, would be of high interest. However, one of the major reasons to apply recommender methods are their capability to handle heterogeneous and sparse data. The application of typical model-based approaches, in turn, is difficult as structure and characteristics of the clinical data at hand, that is, its high degree of heterogeneity and sparsity, would require extensive feature transformation and preparation (handling of missing data, transformation of non-interval-scaled data). As suchlike preparation is complex and will heavily impact the performance of machine-learning algorithms, the usage of such techniques and their comparison to the recommender approaches exceeds the extent of this work. However, future works will address this issue considering the presented clinical data but also using other data in order to yield a comparative assessment of the proposed methods.

## Figures and Tables

**Table 1:** Patient describing attributes.

Attribute	Scale	Range	Availability %
*Patient data*			
Year of birth	Interval	1931–1998	100
Gender	Nominal	1, 2	100
Weight	Ratio	50–165	51.40
Size	Ratio	99–204	35.73
Family status	Dichotomous	0, 1	53.02
Planned child	Nominal	1, 2, 3	8.01
Year of first diagnosis	Interval	1950–2014	89.74
Type of psoriasis	Nominal	1, 2, 3, 4, 5, 6	100
Family anamnesis	Ordinal	1, 2, 3	50.95

*Comorbidities*			
Comorbidity	Nominal	1, 2, 3, ..., 34	—
Status	Ordinal	1, 2, 3	100
Under treatment	Dichotomous	0, 1	100
Disease-free	Dichotomous	0, 1	100

*State of health*			
PASI score	Ratio	0–43	70.57
Self-assessment severity	Ordinal	1, 2, 3, 4, 5	9.45
Development face	Ordinal	1, 2, 3	6.84
Development feet	Ordinal	1, 2, 3	9.81
Development nails	Ordinal	1, 2, 3	20.97
Development hands	Ordinal	1, 2, 3	12.33
Treatment contentedness	Ratio	0–10	10.62

**Table 2:** Therapy describing attributes.

Attribute	Scale	Range	Availability %
Systemic therapy history	Nominal	1, 2, 3, ..., 15	—
Effectiveness	Ordinal	1, 2, 3	23.67
ΔPASI	Ratio	−27–18	42
Adverse effect	Dichotomous	0, 1	100

Systemic therapy	Nominal	1, 2, 3, ..., 15	—
Effectiveness	Ordinal	1, 2, 3	98.43
ΔPASI	Ratio	−27–18	42
Adverse effect	Dichotomous	0, 1	100

**Table 3:** Outcome-driven evaluation definitions.

	Good outcome	Bad outcome
Recommendations compliant	*TP*	*FP*
Recommendations not compliant	*FN*	*TN*
